# Adverse Childhood Experiences of Children Adopted from Care: The Importance of Adoptive Parental Warmth for Future Child Adjustment

**DOI:** 10.3390/ijerph16122212

**Published:** 2019-06-22

**Authors:** Rebecca E. Anthony, Amy L. Paine, Katherine H. Shelton

**Affiliations:** 1 Centre for the Development and Evaluation of Complex Interventions for Public Health Improvement (DECIPHer), School of Social Sciences, Cardiff University, 1-3 Museum Place, Cardiff CF10 3BD, UK; 2 School of Psychology, Cardiff University, Tower Building, 70 Park Place, Cardiff CF10 3AT, UK; paineAL@cardiff.ac.uk (A.L.P.); sheltonKH1@cardiff.ac.uk (K.H.S.)

**Keywords:** adverse childhood experiences (ACEs), adoption, looked after, parental warmth, mental health, child adjustment

## Abstract

We investigated the relationship between adverse childhood experiences (ACEs) and children’s internalising symptoms and externalising problems in the Wales Adoption Cohort Study, a prospective longitudinal study that used case file records (*n* = 374) for a sample of British children adopted from care (*M* = 2 years, 55% male). Parents (*n* = 96) completed questionnaires at 3–5 months, 15–17 months, and 31–33 months post-placement. We hypothesised that: (1) children adopted from care would have experienced more ACEs than children in the general population; (2) the number of ACEs would be associated with higher internalising symptom and externalising problem scores; and (3) adoptive parental warmth would moderate the relationship between ACEs and post-placement internalising symptoms and externalising problems. Nearly half (42%) of the children experienced four or more ACEs. Internalising symptoms and externalising problems were significantly higher than the UK general population. The number of ACEs was associated with internalising symptoms 3 years post-adoptive placement but this relationship was moderated by adoptive parental warmth. This study profiles the experiences and characteristics of a national sample of adopted children and highlights the potential importance of parent warmth as a factor that ameliorates the impact of ACEs on poor child outcomes.

## 1. Introduction

It is well established that adverse childhood experiences (ACEs), such as abuse and neglect, are negatively associated with numerous physical, social, emotional, and behavioural problems that can persist into later life [1,2,3]. However, there remains a paucity of studies that have examined the consequences of ACEs in high-risk samples most likely to have experienced a higher number and variety of early adverse experiences [4], particularly within samples of looked after children and young people [5]. In some exceptions, from samples of children from foster care and with child protection or welfare involvement, researchers have identified an alarming majority were reported to have experienced multiple ACEs [6,7,8], and that there appears to be a dose–response relationship between cumulative ACEs and a greater number of mental and physical health problems [8,9].

### 1.1. Adoption

For the most at-risk children, adoption from care is a permanent care option for children who cannot live with their birth parents. Current UK policy favours achieving permanence within a permanent family setting, with a particular focus on adoption [10]. Research has shown that children adopted from care generally fare better than those who remain in foster care [11]. Adoption provides lifetime relationships with adoptive parents, siblings, and extended families [12] and adoptees generally experience a greater sense of belonging in their adoptive homes [13,14]. However, adoption has been described as a highly charged controversial public intervention [15] due to the radical discontinuity of the child’s relationships with their birth family.

There is currently little research on the vulnerability of adoptees to ACEs [16] despite a number of studies showing that adopted children demonstrate more emotional and behavioural problems than children in the general population (e.g., [17,18,19]). A recent study of adverse experiences in a cohort of “difficult to place” adoptees placed in Australia over 26 years found that adoptees experienced a high rate of ACEs and other factors known to exacerbate vulnerability, such as placement breakdowns [16]. However, the majority of studies investigating the impact of adversity in adoptive samples have focused on individual adversities, rather than cumulative risk (e.g., [20,21]), despite it being established that ACEs often co-occur [1,22]. One exception [23] applied a cumulative risk model to the understanding of behavioural adjustment among a study of international, domestic/public, and domestic/private adopted children in elementary school in the US. The authors [23] found that cumulative risk was associated with adoptive parent reported behavioural adjustment in middle to late childhood.

However, while such work is informative, studies that focus on US or Australian samples are difficult to translate into the UK context due to the different pre-adoption experiences and processes, and consequently, potentially different post-adoptive child outcomes [24]. Specifically, the vast majority of children adopted in the UK are adopted from the looked after population, rather than internationally [25], and domestic private arrangements are not used [26]. Furthermore, most studies assessing risk in adopted samples have relied upon adoptive parents’ knowledge about their children’s pre-adoptive history (e.g., [27]), which may be flawed as full information is not always available or provided [28]. These issues create an incomplete picture of the extent of adversity experienced by children adopted from the UK care system. This may lead to an underestimation of both the prevalence and impact of adversity on post-adoption psychological health and undermine the case for investing in post-adoption support for families.

It is also well known that the age at which children are removed is a key indicator of their later outcomes [29,30]. Although adopted children vary in terms of their pathways to adoption [31], most children adopted from care will have experienced abuse and/or neglect within their birth family [28]. Thus, children who remain in the care of their birth family for longer are more likely to have experienced a higher number of ACEs for a protracted period of time [32]. Compounding this, early adversity is likely to involve multiple sources of risk that occur during crucial developmental stages [33], which may affect physiology and brain maturation [34,35]. While children who are removed at birth may be spared the impact of ACEs in early childhood, they are still vulnerable in terms of their care experience (i.e., repeated separations from caregivers and unstable living arrangements, their genetic history [36], and the higher likelihood of exposure to prenatal adversity, such as drug and alcohol abuse [37]). Therefore, any investigation of the impact of ACEs must take the age at which children were placed with their adoptive family into account.

### 1.2. Parental Warmth as a Moderator of the Impact of ACEs

The association between parenting dimensions and styles and child externalising problems and internalising symptoms are well established [38,39,40]. Positive dimensions of parenting (i.e., characterised as warm, sensitive and responsive) are associated with lower levels of child emotional and behavioural problems [41]. In terms of parenting practices, parental warmth specifically has been shown to be a protective factor for children’s adjustment in a variety of circumstances and across different cultural contexts [42,43,44,45,46]. Children are most likely to thrive in an environment where toxic adversity is minimised [47] and protective factors, such as warm parenting, are enhanced [48]. Warm, sensitive and responsive parenting been found to attenuate the direct effect of adversity on children’s internalising symptoms and externalising problems [49]. Parental warmth may be positively linked to children’s mental health through its effects on the development of children’s emotion regulation and conflict management skills [43,50].

Adoption is described as an intervention that may ameliorate the negative impact of early adversity in childhood via the nurturing caregiving environment offered by adoptive parents [34,47,48]. Specifically, warm parenting is considered one of the most robust protective factors of adopted child adjustment [42]. Within adoption research, there is a small but growing body of evidence that parent–child relationship quality in adoptive families may nurture healthy development. Aspects of adoptive parenting shown to improve children’s behaviour problems and well-being include child-centeredness (e.g., expressed warmth toward their children) [23,45,49], emotional availability [51], and perceived quality of adoptive family relationships [29]. Given that many adoptive families in the UK (especially those who adopted children aged 4 and over) call for more help to strengthen family relationships [52], further work that identifies pathways to better child outcomes is warranted.

### 1.3. The Present Study

There is a dearth of research investigating pre-adoption adversity and its association with children’s adjustment in samples of children adopted from care. Our study takes a unique approach collecting information on pre-adoption experiences using case file records for a national cohort sample of children adopted from care over a 1 year period. We investigated the association with later mental health using a subsample of adoptive families followed prospectively. Understanding what helps adoptee recovery is crucial to promote the well-being of children adopted from care and to support adoptive families. Building on these gaps in the literature, our study aims to investigate: (1) the number and categories of ACEs in a sample of children adopted from care in Wales; (2) associations between adversity and child internalising symptoms and externalising problems; and (3) the moderating effect of warm parenting on associations between the number of ACEs and the child later internalising symptoms and externalising problems. We hypothesised that: (1) children adopted from care would have experienced more ACEs than children in the general population; (2) the number of ACEs would be associated with higher internalising symptom and externalising problem scores; and (3) adoptive parental warmth would moderate the relationship between pre-adoption ACEs and post-placement internalising symptoms and externalising problems.

## 2. Materials and Methods

### 2.1. Study Design

We used data from the Wales Adoption Cohort Study (WACS) [53], a prospective, longitudinal cohort study of a national sample of children placed for adoption from care in Wales. The study used a mixed-methods approach, with data drawn from case file records (*n* = 374) and questionnaires to adoptive parents (*n* = 96).

### 2.2. Ethical Considerations

A multi-disciplinary advisory group for the study provided valuable guidance for developing best practice with respect to the ethics pertaining to safeguarding and data protection. Initial permission was obtained from the Welsh Government to access the local authority data. We then approached and consulted with the heads of Children’s Services Group and then senior adoption managers across Wales to secure their approval to contact social work teams and access records. As the research team had no access to the details of those adoptive families eligible for participation in the study, local authority (LA) social work teams sent out letters on our behalf to prospective families. Families who wanted to take part in the study contacted the research team directly. All subjects gave their informed consent for inclusion before they participated in the study. The study was conducted in accordance with the Declaration of Helsinki, and ethical permission for the study was granted by the Research Ethics Committee at Cardiff University (SREC/1226).

### 2.3. Data Collection

Case file data: Three-hundred-and-seventy-four child adoption reports (CARs; previously known as the child assessment report for adoption) were reviewed. The sample comprised a full cohort of all children placed for adoption by every local authority in Wales between 01 July 2014 and 31 July 2015. The CARs contain the information that local authorities must include when reporting on children put forward for adoption, as set out in the Adoption Agencies (Wales) Regulations (2005). Four research assistants (all female) with backgrounds in psychology (*n* = 2) and social work (*n* = 2) carried out the data collection across Wales, with the lead author responsible for 83% (*n* = 309) of the data collection. The researchers worked on site at the local authority offices and entered data directly into an SPSS database. About two-thirds of the CARs reviewed were in electronic format, whilst the remainder were hard copies. More than 250 discrete pieces of information were sought from each CAR record. Reliability checks involved researchers blind coding subsequent checking of five records to ensure a common understanding of and consistent approach to coding. The strengths and limitations of using case file records for research purposes have been previously discussed [53,54].

Questionnaire data: All adoptive parents of children placed for adoption by every LA in Wales between 01 July 2014 and 31 July 2015 were invited to take part in the study. Adoptive parents completed a questionnaire at 3 time points: 3–5 months into the adoptive placement, 15–17 months into placement and 31–33 months into placement (T1, T2 and T3, respectively). Of the original 96 respondents recruited at T1, 84% participated at T2 (*n* = 81) and 74% at T3 (*n* = 71). Demographic differences (gender, age, relationship status, education and income) and child behaviour symptoms due to the attrition between T1 and T3 were explored using *t*-tests and chi-square tests. There were no statistically significant differences for those families who participated at T1 compared to those who remained in the study at T3 (all *ps* > 0.05).

### 2.4. Participants

For the CAR sample which included all children placed for adoption in Wales over a 1 year period, the majority were placed for adoption aged 12 months (range 0–9 years, *M* = 2 years), just over half (55%) of the children were male and the majority were white British (93%). For the parents of the longitudinal sample, the majority of respondents were female (92%, *n* = 87, 1 person did not respond). The mean age was 41 (ranged from 23 to 62) and the majority (99%, *n* = 94) were white British. The majority of respondents were in a heterosexual relationship (82%, *n* = 79), a small percentage (5%, *n* = 5) were in a same sex relationship and 13% (*n* = 12) were single adopters. Most of the sample (70%, *n* = 67) adopted 1 child, 27% (*n* = 26) adopted 2 children together, and 3% (*n* = 3) adopted a group of 3. The gross family income and education levels were substantially higher than the UK average according to a comparison with ONS data [55], where 13% earned more than £75,000 per year and 37% had postgraduate degrees.

### 2.5. Measures

#### 2.5.1. Adversity

Adverse childhood experiences (ACEs): Ten categories of ACEs were coded to closely match the original Felitti (1998) [1] study. These included: childhood abuse (emotional, physical, and sexual); neglect and household dysfunction (growing up with domestic violence, parental separation, substance abuse, alcohol abuse, mental illness, and incarceration). Table S1 (Supplementary Materials) presents a list of ACEs definitions from the original study [1] and this study’s equivalent. Variables were coded as either absent (0) or present (1). If there were recorded allegations of maltreatment or household dysfunction, these were coded as present based on previous findings that children with alleged abuse and substantiated abuse are at a similarly increased risk for mental health and behavioural consequences [56]. The decision was taken that if information was not recorded on the CAR for any ACE variable it was coded as “absent” due to the social work caseworkers’ instruction to provide detailed information for all aspects of the child’s home life for the courts. Exposure to ACEs were captured in the ACEs count; a simple count of exposure to each of the 10 different types of adverse experiences during the child’s time spent with birth parents, before being placed for adoption. Exposure to any ACE category counted as 1 “point” towards the overall score.

#### 2.5.2. Age Placed for Adoption

The age of children when they were placed for adoption (in years) was calculated by subtracting the child’s birth date from the date they were placed for adoption.

#### 2.5.3. Child Internalising Symptoms and Externalising Problems

Adoptive parents completed 1 of 2 versions of the Strengths and Difficulties Questionnaire (SDQ) [57]. One version is for 2–4 year olds and another version for 4–18 year olds. The SDQ consists of five subscales, measuring emotional symptoms, conduct problems, hyperactivity-inattention, peer relationship problems, and prosocial behaviours. Each subscale contains 5 items on a three-point Likert-type scale to measure to what extent a symptom, e.g., “considerate of other people’s feelings” applied to their child’s behaviour over the last six months, using the options “Not true”, “Somewhat true”, or “Certainly true”, with scores ranging from 0–10. A higher score is indicative of more problems for all subscales, except for the prosocial scale, where higher scores correspond to strengths in prosocial behaviour. The 2 versions are identical, except that in the younger version, the item on reflectiveness is re-phrased and 2 items on antisocial behaviour are replaced by items on oppositionality. The SDQ is a psychometrically sound measure of overall child mental health problems in studies from around the world [58,59]. Reliability, validity, internal consistency, test–retest reliability after 4 to 6 months, and interrater agreement for the SDQ are satisfactory [60]. The SDQ is also deemed an appropriate screening tool for detection of emotional, behavioural, and concentration problems among looked-after children [61].

Within our study, the hyperactivity and prosocial behaviours scales had acceptable to good levels of internal consistency across the 3 time points (hyperactivity: α = 0.737 to 0.829; prosocial behaviour: α = 0.713 to 0.744; emotional problems: α = 0.664 to 0.769; conduct problems: α = 0.670 to 0.675). The peer problems scale had Cronbach’s alphas ranging from 0.463 to 0.590 across T1–T3. We used internalising symptoms and externalising behaviour problems as outcome variables as the key outcomes in the study. The SDQ externalising problems score is the sum of the conduct and hyperactivity scales and ranges from 0 to 20. The SDQ internalising symptoms score is the sum of the emotional and peer problems scales and ranges from 0 to 20.

#### 2.5.4. Parent-to-Child Warmth

At the T2 questionnaire follow-ups, parents (88%, *n* = 71 adoptive mothers, 12%, *n* = 10) adoptive fathers) completed the warmth scale of the Iowa Family Interaction Rating Scales [62] (parent-to-child). The measure contains 6 items on a 7 point scale ranging from “never” to “always” with high scores indicating greater warmth (e.g., “Let him/her know you really care about him/her,” “Act loving and affectionate towards him/her,” and “Tell him/her you love him/her”). Internal consistency estimates were acceptable (α = 0.938 to 0.948).

#### 2.5.5. Missing Data

CAR sample: We had complete information for pre-placement adversity variables. Case file data used to index early adversity *and* parent-reported assessment of the child mental health symptom severity were available for: *n* = 84 at T1, *n* = 71 at T2 and *n* = 62 at T3. There were eight cases where the questionnaires returned by adoptive parents could not be matched with a CAR record. Missingness in the questionnaire data was handled in MPlus using full-information maximum-likelihood (FIML) estimation based on the assumption that data are missing at random. Little’s test indicated that the data were completely missing at random for T2 parental warmth, *χ*^2^(10) = 11.73, *p* > 0.05, T3 internalising symptoms, *χ*^2^(26) = 32.88, *p* > 0.05 and T3 externalising problems, *χ*^2^ (36) = 27.12, *p* > 0.05. Full-information maximum-likelihood uses all of the available information for each participant rather than deleting participants or imputing values [63].

#### 2.5.6. Analysis

We first describe the number of pre-placement ACEs in both the CAR sample and the longitudinal subsample to facilitate comparability with other studies. Focusing on the longitudinal subsample, SDQ scores were then compared to community sample populations using one-sample *t*-tests. Correlation matrices were used to examine relationships between adversity and mental health symptom severity. In cases where variables were not normally distributed, Spearman’s rank–order correlations were used to assess relationships. Due to the large number of white British children in our sample, ethnicity was dichotomised into “white” and “any other ethnicity” to ensure anonymity.

The final analysis investigating associations between adversity and later child mental health (and the potential moderating role of adoptive parent warmth) was conducted using linear regression and simple slopes analysis in Mplus version 7.11 [64]. Two separate multiple regression models were tested to predict the main and joint effects of ACEs and warm adoptive parenting on children’s internalising symptoms and externalising problems, whilst controlling for children’s age at placement. A power analysis demonstrated that the sample was adequately powered to detect small to moderate effect sizes (*d* = 0.25) with the probability of making a type one error being 0.05 and power being 0.80 (required *n* = 53). Point-biserial correlations showed no statistically significant correlations between gender or ethnicity and SDQ symptom scores, thus, due to the sample size restrictions, these variables were not retained for further analysis, as recommended [65]. The variance inflation factor value (1.21) and tolerance value (0.83) suggested the absence of multicollinearity [66]. Models were estimated using the robust maximum likelihood estimator (MLR), which corrects fit indices and standard errors to account for non-normality in the data, equivalent to the Yuan–Bentler T2*test statistic [67].

## 3. Results

### 3.1. Description of Pre-Placement Experiences

Over a third of children (41% CAR sample, 35% longitudinal subsample) were placed into local authority care at birth. In the CAR sample, children spent a mean of 415 days with their birth parents (range 0–2344) and 530 days in care (range 0–2532). In the longitudinal subsample, children spent a mean of 523 days (range 0–2344) with their birth parents and 540 (range 0–1401) days in care. In both the CAR and longitudinal samples, nearly two-thirds of children (65%) had one foster placement before being placed for adoption (range 0–8 in the CAR sample and 0–7 in the longitudinal, *M* = 2). In the CAR sample, the majority were placed for adoption aged one, whereas in the longitudinal sample, the majority were placed for adoption aged zero (range 0–9, *M* = 2 in both samples). Children in the longitudinal sample were slightly older than the full CAR sample because we asked adoptive parents of sibling groups to comment on the eldest child placed for adoption. Independent samples’ *t*-tests and chi-square tests showed no significant differences between the CAR sample and longitudinal subsample in their pre-placement characteristics (all *p*s > 0.05).

**Figure 1:**
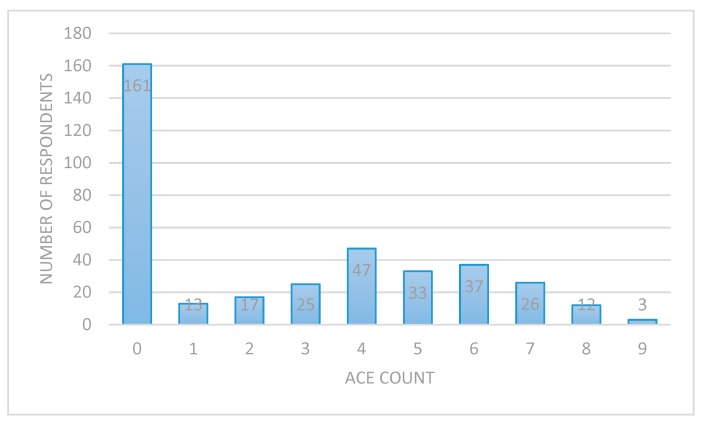
Number of adverse life experiences (ACEs) children experienced in the care of their birth parents (*n* = 374, child adoption reports [CAR] sample).

Figure 1 shows that the number of ACEs ranged from zero through to nine. The median number of ACEs the children in our sample had encountered was two, and the mean was three.

Table 1 shows that pre-adoption exposure to adversity was common, with over half (54%) of the sample experiencing neglect, 37% being exposed to a domestic violence and 34% being exposed to a parent who abused drugs. No differences were detected between the number of ACEs in the CAR sample and in the longitudinal subsample (*p* > 0.05).

### 3.2. Child Internalising Symptoms and Externalising Problems

One-sample *t*-tests with a Bonferroni corrected alpha of 0.01 were used to determine whether the children in our sample had significantly different scores on the parent reported SDQ total problems score, compared to a nationally representative sample of 10,298 UK pupils aged 5–15 [68] and a nationally representative sample of children looked after aged 5–16 (*n* = 1391 [69]. Table 2 shows elevated difficulties on the SDQ total scales, and at all time points scores were significantly higher than the UK general population. In contrast, at T1, children’s SDQ scores were statistically significantly lower than looked after children living with their parents (*t*(57) = −2.997, *p* < 0.01) and those in residential care (*t*(57) = −2.997, *p* < 0.01). However, at T2 and T3, our longitudinal sample scores were statistically significantly lower than children in all other types of care.

In addition to comparing mean scores, scores were classified into cut-offs based on normative samples [57]. Table 3 shows that for the total difficulties subscale, 21% (*n* = 12) were classified as having “very high” scores compared to the 5% in the normative population. The largest differences between our study sample and normative comparison group can be seen in the hyperactivity and prosocial subscales at T1, where approximately half (48 to 51%) were classified as being within the “normal” range, compared to 80% of the normative population.

### 3.3. The impact of ACEs on Child Internalising Symptoms and Externalising Problems: The Moderating Effect of Parental Warmth

Table 4 shows correlations between variables of interest. There was a strong positive association between ACE count and age placed for adoption, *r*(373) = 0.615, *p* < 0.001. There were positive associations between age placed and internalising symptoms at T3, *r*_s_(62) = 0.381, *p* < 0.01. Age placed was also associated with reduced parent to child warmth, *r*_s_ (72) = −0.45, *p* < 0.01. The ACE count was associated with increased internalising symptoms at T3, *r*_s_ (62) = 0.32, *p* < 0.05, and reduced parent to child warmth, *r*_s_ (72) = −0.29, *p* < 0.05. The T2 parent to child warmth was associated with fewer child internalising symptoms, *r*_s_ (68) = −0.43, *p* < 0.01 and externalising problems, *r*_s_ (68) = −0.39, *p* < 0.01 at T3.

#### 3.3.1. Externalising Problems

The full model established that neither age placed or ACE count predicted externalising problems at T3 (0.029, Est./S.E. = −0.113, *p* = 0.910; 0.114, Est./S.E. = 0.567, *p* = 0.570), respectively. Parent to child warmth at T2 did not predict externalising behaviour problems at T3 (−0.171, Est./S.E. = −1.505, *p* = 0.132). The interaction between ACE count and T2 parental warmth on T3 externalising problems was also non-significant (0.006, Est./S.E. = 0.186, *p* = 0.853), see Table 5.

#### 3.3.2. Internalising Symptoms

**Figure 2:**
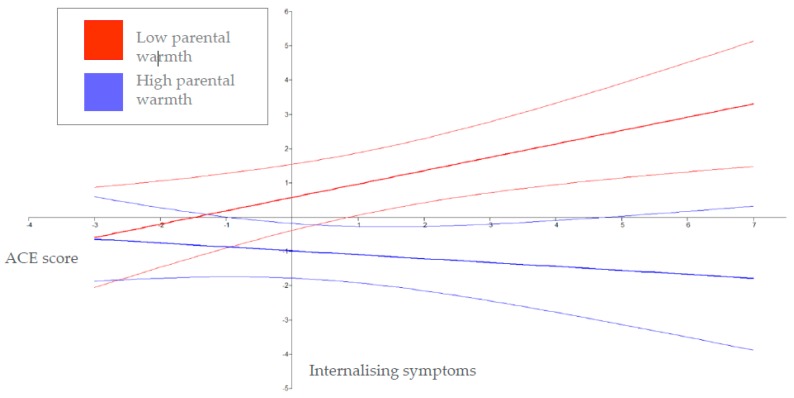
The moderating role of parental warmth (±1 SD) in the relationship between ACE count and T3 internalising problems (*n* = 62). Note: The faded lines above and below low and high warmth show the 95% confidence bands.

The full model established that neither age placed or ACE count predicted internalising behaviour symptoms at T3 (0.197, Est./S.E. = 0.967, *p* = 0.334; 0.026, Est./S.E. = 0.178, *p* = 0.859), respectively. Parent to child warmth at T2 predicted fewer internalising symptoms at T3 (−0.144, Est./S.E. = −1.965, *p* = 0.049). Furthermore, there was an interaction between ACE count and T2 parental warmth on T3 internalising symptoms (−0.059, Est./S.E. = −2.925, *p* = 0.003). Within this model, approximately 18% of the variability in T3 internalising behaviours (*R*^2^ = 0.181) was explained. As indicated by the significant interaction term, the nature of the relationship between ACE count and T3 child internalising symptoms was moderated by adoptive parent warmth to the child. Figure 2 shows the effect of ACE count on children’s T3 internalising symptoms at 1 SD below and 1 SD above the zero mean of parental warmth measured at T2, plotted over the full range of ACE count and internalising symptoms. ACE count was unrelated to internalising symptoms at higher levels of parental warmth (−0.11, Est./S.E. = −0.78, *p* =.440), although children who experienced higher levels of maternal warmth had lower internalising problems. In contrast, ACE count was significantly positively related to internalising symptoms at lower levels of parental warmth (0.390, Est./S.E. = 2.802, *p* = 0.005, Table 5).

## 4. Discussion

The present study adds to a relatively small literature on the impact of ACEs on internalising symptoms and externalising problems in adopted children. In line with our hypotheses, we found that children placed for adoption in Wales have experienced many adverse experiences prior to placement. Over two-fifths of the children in our CAR sample had experienced four or more ACEs, which is a similar profile to other studies with looked after children [6,8], and substantially more than the estimated 14% of the adult general population in Wales [70]. In comparison to general population [57,68] and children looked after samples [69], children in our sample were reported by parents as having more difficulties (as rated with the SDQ). This is in line with extensive research that has investigated the mental health of children looked after [71] and literature showing that adopted children have high levels of problem behaviours (e.g., [17,32,69,72]). Our findings support the need for effective interventions that can meet the needs of adopted children with respect to their mental health [73].

The ACE count was only modestly associated with internalising problems at T3 (31 to 33 months post-placement) and in contrast to our hypothesis, was not associated with externalising problems. Our findings may reflect the effectiveness of a nurturing environment [74], supporting the view that, “one is not doomed to a poor developmental outcome as a function of early adversity” ([75], p.2). For example, previous studies have found no relationship between early risk and later health outcomes for children adopted privately as infants [76] or those children who experienced adversity only early in life (i.e., before aged six) [77]. However, it is important to consider that children can appear well following disclosure, but may become more symptomatic later in development [78]. Thus, these findings may be due to the young age of our sample, where the full extent to which associations between pre-adoption adversity, post-adoption family climate and mental health may be yet to unfold.

Our results support previous studies suggesting that warm parenting is associated with lower levels of internalising and externalising problems [49], extending findings to a group of UK children adopted from care. Children who experienced less adversity (e.g., a lower number of ACEs) and whose adopted parents reported higher expressions of parent to child warmth, had the lowest levels of internalising problems. In line with our hypothesis, we demonstrated that low warmth moderated the relationship between ACE and internalising symptoms, such that those children with a higher number of ACEs and adopted parents reports of lower parent to child warmth had higher internalising symptoms. These findings underscore the need to support adoptive parents to forge mutually rewarding relationships with children before and after placements commence. One possibility is to foster these relationships by including parents in therapeutic work with children exposed to adversity, an approach associated with superior outcomes relative to interventions only working with the child [79].

### 4.1. Strengths

Most studies measuring ACEs rely on retrospective adult reports (e.g., [1,70]). Although prior work suggests that reports of ACEs are reliable [80] there is evidence that adult recall of childhood abuse experiences is poor [81]. The accuracy of retrospective reports of childhood events can be influenced by any number of factors, including age when an adversity was encountered [82], memory problems, a desire to protect parents, a desire to deny or forget the past, or methodological issues such as lack of rapport with the interviewer [83]. Additionally, most studies assess adversity at the same time as psychopathology, increasing the chance that recall is biased by current psychological state [84]. Most studies assessing risk in adopted samples have relied upon adoptive parent’s second-hand information about their child’s pre-adoptive history, which may be inaccurate.

Our study adopted a different approach in a marked departure from existing work in this area. We estimated adversity using independent social worker reports of the children’s experiences before being placed for adoption, which yielded information about the characteristics and pre-placement experiences of a national sample of children recently placed for adoption in the UK. Furthermore, this study used a prospective longitudinal design, adding to the relatively few prospective, longitudinal studies on the psychological consequences of child abuse [85].

### 4.2. Limitations

Although sizable for a study of children adopted from foster care and adequately powered, the sample size was small; thus, we were limited in our analysis and caution must be exercised in interpreting results [86]. The present study was limited to the use of parent reports of child internalising symptoms and externalising problems due to the young age of the children when placed for adoption, which precluded using teacher or child reports of difficulties. Whilst it has been suggested that adoptive parents may pay greater attention to their children’s adjustment than birth parents [17], a recent study refuted this [87]. However, we speculate that adoptive parents may be less able to accurately report on their child’s externalising problems and internalising symptoms early on in the child’s placement as parents are likely to be getting to know their children; this may account for the lack of association detected between ACEs and children’s externalising problems.

As information about childhood adversity was derived from independent reports, we believe it to be more accurate than second-hand adoptive parent reports. However, it is important to note that some ACE variables such as sexual abuse, where children will often only divulge information at an older age [88], may be underestimated. In addition, case file records will inevitably miss some, potentially important, information about a child’s life [89].

Using an ACE approach has been criticised due to the over-simplifying very complex and challenging issues and leading to a substantial loss of information (including the severity, timing and length of exposure to adversity, as well as who perpetrated the adversity). Whilst taking an “ACE count” approach to contextualising children’s pre-adoption experience is helpful to aid comparisons, the approach likely represents an underestimate of the cumulative effects of adversity in the context of children adopted from care. For example, we could not index each child having experienced the loss of relationships with their birth family, foster parents, their home and their possessions. In addition, prenatal and children’s experience in the period between being removed from birth parents and placed for adoption (i.e., moves between foster parents and changes to their social and physical environment, e.g., school) are not taken into account. Furthermore, other aspects of the adoptive home, such as adoptive parents’ socioeconomic status, their mental health and sibling relationship quality may be important in moderating the relationship between pre-adoption adversity and post-adoption outcomes.

## 5. Conclusions

This study reveals the social conditions in which parents and children live and highlights the multiple stressors that children placed for adoption in the UK have experienced. The mental health of children in our sample was significantly worse than the UK general population at all time points, highlighting the need for support services for families who adopt children from care. The failure to meet the complex needs of children who have experienced adversity has been recognised and identified as a public health concern [90]. The “ACE count” was only associated with internalising symptoms at the 31 to 33 month assessment. Therefore, we suggest that whilst policy, training and service design should be “informed” by ACE, other aspects of a child’s history, such as length of exposure to adversity, time in care, number of moves, sibling relationship quality, (for children placed together) and contact (reflecting separation from significant others), may provide a broader picture of potential risk. Furthermore, screening for adversity only has any real benefit to a child when there are established interventions that mitigate some potential harmful outcome that the screening identifies [91]. Our findings highlight the impact of the caregiving environment for child psychopathology. We suggest that interventions directed at enabling warm parenting are a promising avenue for improving the emotional problems of adopted children.

## Figures and Tables

**Table 1:** Child ACEs in the CAR and longitudinal subsample.

Adverse Childhood Experiences (ACEs)	CAR Sample (*n* = 374)	Longitudinal Subsample (*n* = 84)
Emotional abuse	85 (23)	20 (24)
Physical abuse	70 (19)	19 (23)
Sexual abuse	14 (4)	5 (6)
Neglect	203 (54)	45 (54)
Parental separation	113 (30)	24 (29)
Domestic violence	138 (37)	34 (31)
Parent mental illness	118 (32)	31 (37)
Parent alcohol abuse	96 (26)	20 (24)
Parent drug abuse	126 (34)	27 (32)
Parent incarceration	74 (20)	19 (23)
ACE “score” (M, SD)	2.68 (2.75)	2.65 (2.82)

Note: Descriptive shown as number (%) unless otherwise stated.

**Table 2:** Comparison of SDQ total scores between general UK and looked after populations and the longitudinal subsample in the present study.

Samples and Subpopulations	*n*	SDQ Total Difficulty Score, Mean (95% CI)
UK general population	10,298	8.4
Looked after children		
Foster care	781	15.3 (14.7–15.8)
At risk children living with natural parents	190	16.2 (15.0–17.3)
Kinship care	165	12.2 (11.0–13.4)
Residential care	255	20.0 (19.1–20.8)
Wales Adoption Cohort Study		
Time 1	58	13.6 (11.87–15.43)
Time 2	76	10.6 (9.31–11.81)
Time 3	70	10.8 (9.40–12.17)

Note: SDQ total scores were retrieved from reference [62] for the UK general population and reference [63] for all “children looked after” samples.

**Table 3:** Categorical SDQ scores of the longitudinal subsample.

	Average	Slightly Raised	High	Very High	Average	Slightly Raised	High	Very High	Average	Slightly Raised	High	Very High
Normative sample	80%	10%	5%	5%	80%	10%	5%	5%	80%	10%	5%	5%
	Time 1 *n* (%)				Time 2 *n* (%)				Time 3 *n* (%)			
Total difficulties	34 (59)	7 (12)	5 (9)	12 (21)	50 (66)	15 (20)	6 (8)	5 (7)	48 (72)	6 (9)	7 (10)	6 (9)
Emotional problems	39 (67)	5 (9)	7 (12)	7 (12)	55 (72)	11 (15)	5 (7)	5 (7)	51 (76)	3 (5)	7 (10)	6 (9)
Conduct problems	36 (62)	6 (10)	7 (12)	9 (16)	60 (79)	5 (7)	5 (7)	6 (8)	43 (64)	10 (15)	8 (12)	6 (9)
Hyperactivity	29 (51)	13 (23)	3 (5)	12 (21)	50 (66)	13 (17)	5 (7)	8 (11)	47 (70)	6 (9)	5 (8)	9 (13)
Peer problems	38 (67)	10 (18)	3 (5)	6 (11)	54 (71)	13 (17)	4 (5)	5 (7)	47 (70)	5 (8)	9 (13)	6 (9)
Prosocial	28 (48)	8 (14)	10 (17)	12 (21)	46 (60)	5 (7)	10 (13)	16 (21)	37 (55)	8 (12)	11 (16)	11 (16)

Note: Percentages for normative sample were retrieved from Reference [56].

**Table 4:** Inter-correlations between variables of interest.

Measure		1	2	3	4	5	6
1	Child gender	-					
2	Child age at placement	−0.07 (373)	-				
3	ACE count	−0.09 (374)	0.62 ** (373)	-			
4	T3 child externalising	−0.01 (62)	0.15 (62)	0.14 (62)	-		
5	T3 child internalising	−0.08 (62)	0.36 ** (62)	0.26 * (62)	0.48 ** (70)	-	
6	T2 parent warmth	−0.12 (72)	−0.45 ** (72)	−0.29 ** (72)	−0.39 ** (69)	−0.43 ** (69)	-
Mean		0.52	2.32	2.65	6.98	3.80	38.17
(SD)		(50)	(2.23)	(2.82)	(3.61)	(3.07)	(4.45)

Note: * *p* < 0.05, ** *p* < 0.01, the number of participants is shown in brackets.

**Table 5:** Regression coefficients testing the direct and interaction effects of ACE and adoptive parental warmth on children’s internalising and externalising problems (*n* = 62).

Variables	T3 externalising Problems			T3 internalising Problems		
	B	SE	β	B	SE	β
Age placed	−0.03	0.26	−0.02	0.20	0.20	0.16
ACE count	0.11	0.20	0.09	0.03	0.15	0.03
T2 parental warmth	−0.17	0.11	−0.22	−0.14 **	0.07	−0.22
ACE × parental warmth	0.01	0.04	0.02	−0.06 **	0.02	−0.24 *

Note: * *p* < 0.05, ** *p* < 0.01.

## Supplementary Materials

The following are available online at https://www.mdpi.com/1660-4601/16/12/2212/s1, Table S1: List of original ACEs definitions and WACS study equivalent.
