# Supplementary material for: Adverse Childhood Experiences of Children Adopted from Care: The Importance of Adoptive Parental Warmth for Future Child Adjustment
Source: Int J Environ Res Public Health. 2019 Jun 22;16(12):2212. doi: 10.3390/ijerph16122212 (PMC6617038; doi:10.3390/ijerph16122212)
Supplement: Supplementary file 1 [file ijerph-16-02212-s001.zip › ijerph-498499-supplementary final/Table S1.docx]

**Table S1.** List of original ACEs definitions and WACS study equivalent.

| ACEs construct | ACEs Definition (Felitti et al., 1998) | Social Work records |
| --- | --- | --- |
| Emotional abuse | Often or very often a parent or other adult in the household swore at you, insulted you, or put you down and sometimes, often or very often acted in a way that made you think that you might be physically hurt. | Caseworker report of substantiated or allegations of emotional abuse based on evidence of psychological aggression, such as threatening the child or calling him/her names. |
| Physical abuse | Sometimes, often, or very often a parent or other adult in the household pushed you, grabbed you, slapped you, threw something at you, or ever hit you so hard that you had marks or were injured. | Caseworker report of substantiated or allegations of physical abuse based on evidence of severe assault or physical abuse, such as shaking an infant or hitting an older child. |
| Sexual abuse | An adult or person at least 5 years older ever touched or fondled you in a sexual way, or had you touch their body in a sexual way, or attempted oral, anal, or vaginal intercourse with you or had oral, anal, or vaginal intercourse with you. | Caseworker report of substantiated or allegations of sexual abuse based on evidence of sexual abuse or forced sex reported by the child or parent. |
| Neglect | Respondents were asked whether they had enough to eat, if their parents’ alcohol drinking interfered with their care, if they ever wore dirty clothes, and if someone was available to take them to the doctor.  Respondents were asked whether their families made them feel special and loved, and were asked if their family was a source of strength, support, and protection. | Caseworker report of substantiated or allegations of child neglect based on evidence of parental failure to supervise, protect or provide for the child. |
| Domestic violence | Mother or stepmother was sometimes, often, or very often pushed, grabbed, slapped, or had something thrown at her and/or sometimes often, or very often kicked, bitten, hit with a fist, or hit with something hard, or ever repeatedly hit over at least a few minutes or ever threatened or hurt by a knife or gun. | Caseworker report of domestic violence based on evidence such as slapping, hitting, or kicking (includes both male and female caregivers who reported domestic violence). |
| Parental separation | Parents were ever separated or divorced. | Caseworker report of parents ever separated or divorced. |
| Mental illness | A household member was depressed or mentally ill or a household member attempted suicide. | Caseworker report of either birth parent having mental health issues. |
| Substance/drug abuse | Lived with anyone who was a problem drinker or alcoholic or lived with anyone who used street drugs. | Caseworker report of evidence of alcohol or drug abuse by either parent. |
| Incarceration | A household member went to prison. | Caseworker report of evidence of either parent spending time in prison. |
